# Linking physical and social environments with mental health in old age: a multisensor approach for continuous real-life ecological and emotional assessment

**DOI:** 10.1136/jech-2020-214274

**Published:** 2020-11-04

**Authors:** Amanda Fernandes, Frank J Van Lenthe, Julie Vallée, Cedric Sueur, Basile Chaix

**Affiliations:** 1 INSERM, Institut Pierre Louis d’Épidémiologie et de Santé Publique, Nemesis Research Team, Sorbonne Université, Paris, France; 2 Department of Public Health, Erasmus University Medical Center, Rotterdam, Netherlands; 3 Department of Human Geography and Spatial Planning, Utrecht University, Utrecht, Netherlands; 4 UMR Géographie-cités, Centre National de la Recherche Scientifique, Paris, France; 5 CNRS, IPHC UMR 7178, Université de Strasbourg, Strasbourg, France; 6 Institut Universitaire de France, Paris, France

**Keywords:** Environmental epidemiology, Ageing, Research methods, Mental health, Stress

## Abstract

**Background:**

Urban stress is mentioned as a plausible mechanism leading to chronic stress, which is a risk factor of depression. Yet, an accurate assessment of urban stressors in environmental epidemiology requires new methods. This article discusses methods for the sensor-based continuous assesment of geographic environments, stress and depressive symptoms in older age. We report protocols of the promoting mental well-being and healthy ageing in cities (MINDMAP) and Healthy Aging and Networks in Cities (HANC) studies nested in the RECORD Cohort as a background for a broad discussion about the theoretical foundation and monitoring tools of mobile sensing research in older age. Specifically, these studies allow one to compare how older people with and without depression perceive, navigate and use their environment; and how the built environments, networks of social contacts, and spatial mobility patterns influence the mental health of older people.

**Methods:**

Our research protocol combines (1) Global Positioning System (GPS) and accelerometer tracking and a GPS-based mobility survey to assess participants’ mobility patterns, activity patterns and environmental exposures; (2) proximity detection to assess whether household members are close to each other; (3) ecological momentary assessment to track momentary mood and stress and environmental perceptions; and (4) electrodermal activity for the tentative prediction of stress. Data will be compared within individuals (at different times) and between persons with and without depressive symptoms.

**Conclusion:**

The development of mobile sensing and survey technologies opens an avenue to improve understanding of the role of momentary stressors and resourcing features of residential and non-residential environments for older populations’ mental health. However, validation, privacy and ethical aspects are important issues to consider.

## INTRODUCTION

Depression is a major mental disorder in older urban populations. Symptoms of depression include sadness, anxiety and insomnia.^1^ These emotional threats modulate older people’s experience of and adaptability to the urban environment, potentially impacting their social network, health behaviour and quality of life.^1 2^

The large literature linking environmental characteristics to depressive symptoms has reported potential associations with poor housing quality, lack of green spaces, noise and air pollution but also inconsistent associations with walkability, availability of services and aesthetics.^1 3 4^ It is important to understand the plausible mechanisms underlying these associations to strengthen causal inference.

For people living in cities, there are between-neighbourhood variations in the exposure to daily environmental stressors and restoration opportunities^1 2^ that influence the likelihood of depressive symptom.^1 4^ Urban stressors are mentioned as a plausible mechanism leading over time to a situation of chronic stress, which is a risk factor of depression, including through mid- to long-term biological mechanisms that are not investigated and reviewed here.^1^ However, new methods are required to better capture the real-life exposure to and influence of urban stressors over daily activities.

First, static measures of contextual exposures that assign participants to their residential neighbourhood as if they were permanently exposed to it have been identified as a limitation.^3 5^ People’s activity space was found to modify the strength of associations between residential neighbourhood characteristics and depression.^6^ A recent smartphone-based investigation demonstrated the relevance of areas beyond the residential neighbourhood to the everyday activities of older adults.^7^

Second, a better assessment of exposures and health effects is required not only over space but also over time. Indeed, the personal exposure to urban resources and hazards occurs over space but also in a dynamic way and defined order over time.^8^ For example, cumulative stress (eg, successive stressful exposures) without adequate coping opportunities (eg, restoration in green or blue environments) might lead to depression in vulnerable individuals through biological and psychological mechanisms.^1 9^ Therefore, the sequence of daily events is a key aspect to understand whether urban environments result in stress or restoration for older persons.^8–10^ Another challenge related to the time dimension pertains to the ‘daycourse of place’, that is, the daily variations affecting characteristics of a given place, such as whether services or parks are open, whether a neighbourhood is crowded or not, etc.^11^ Rather than considering places as necessarily frozen over a 24-hour period, future research needs to explicitly consider the characteristics of places at the time of the day where they are visited when assessing momentary environmental exposures.^11^

Third, the geographic dispersion of the social network influences daily mobility, and consequently daily exposures.^12^ Moreover, whether study participants are alone in a place or with friends or other acquaintances is an important aspect of the circumstances of exposure. Such situational factors^13^ could modulate the impact of built environments on stress. For example, spending time in a park might have a different restorative effect if the participant is alone or with friends or pets.^9^

Fourth, most of the evidence about the relationship between stress resulting from environmental stimuli and depression is from laboratory experiments^14 15^ or short controlled experiments.^16^ To achieve external validity, ambulatory assessment of stress has used self-report or interviews.^14^ However, these measurement strategies are vulnerable to reporting bias (eg, depression inducing negative environmental perception) and recall bias,^3^ especially when a retrospective assessment is performed long after the stress episode.^14^

The improvement of mobile sensing, the miniaturisation of biosensors allowing measurements that were only available in laboratory settings and Global Positioning System (GPS) tracking combined with geographic information systems permit to develop accurate studies of the short-term links between environmental exposures and depressive symptoms.^5 14^ The passive ambulatory monitoring of physiological parameters of stress combined with a smartphone survey assessment of stress close from its occurrence should permit to detect which specific features of the urban settings trigger momentary stress over space and time.^13^

This paper discusses the continuous monitoring methodology that was developed to assess real-life environmental exposures, stress and depressive symptoms in older persons, as a background for a broad discussion about the theoretical foundation and monitoring tools for mobile sensing in the environment and mental health research in older age.

## METHODS

### Aims and hypotheses

As part of the MINDMAP^17^ and HANC projects, we designed a multisensor study to investigate the interplay between the social and physical environments and depressive symptoms in older people. In one direction, our goal is to identify the environmental determinants of depressive symptomatology, through both between-participant comparison and within-participant comparison (considering repeated measurements over several days). In the other direction, we aim to compare how depressed and non-depressed participants perceive, navigate in and use their surrounding environment. The latter objective is related to the ‘negative bias’ or propensity of depressed people to perceive everyday life situations and surrounding environments as worse and more stressful than they are.^18^

By integrating mobile sensing tools in a daily-life monitoring design,^19^ the study aims to explore the ‘black box’ linking environments with poor mental health using fine-grained metrics of exposures, health behaviour and mood disaggregated over space and time. More than the description of geographic contexts of behaviours and mood, this MINDMAP and HANC protocol in RECORD is aligned with current investigations of how built environments predict mood and depression severity based on smartphone GPS locations.^20^ Similarly, the project investigates how the sequence of stressful environmental exposures and resourcing contexts along the day explains the within-individual variability in stress and depressive symptomatology along space and time.^9 13^

This approach should allow us to identify the sequence and combination of neighbourhood social and physical exposures^9 10^ and situational predictors (eg, related to the time of day and day of week, weather, accompanying social contacts, activities)^13 21^ that improve or deteriorate mental health of older people. The ultimate aim is to investigate the environmental mechanisms triggering stress reactions, but also the strategies of older people to deal with these stressors and the environmental restoration opportunities available, in order to inform decision makers and develop community-based and targeted interventions.^22^

### Overview of the study protocol

We developed a cross-sectional study of residents of Paris and its metropolitan region, aged 60 years and more, with and without depression (as screened with the Center for Epidemiologic Studies—Depression scale, CES-D-20). The sample is being recruited (with a target of 200 participants) from the second wave of the RECORD Cohort.^23^ Selection criteria include having a score ≥17 on the Mini-Mental State Examination cognitive test, being able to use a smartphone, and living in the Ile-de-France region.

To assess environmental exposures and mood during older people’s everyday life, the MINDMAP and HANC studies in RECORD integrate traditional computer-based surveys, a web-based mapping application (VERITAS),^24^ mobile sensing tools and a GPS-based web mobility survey.^19 25^ Participants are assisted by research staff to fill out a standard computerised questionnaire assessing several domains and the VERITAS web mapping tool based on Google Maps. The latter application permits to report the boundaries of their perceived neighbourhood, to collect on a map usual recreational walking routes, to geocode their regular destinations, to assess with whom they usually spend time at these places and finally to evaluate their entire social network. As detailed in table 1, the 7-day monitoring protocol includes (1) a GPS receiver and an accelerometer worn on the waist on the right side during wake times; (2) another accelerometer worn on the non-dominant wrist to measure sleep patterns; (3) smartphone-based ecological momentary assessment (EMA) to survey environmental perceptions and anxious and depressive mood through the Eco-Emo Tracker application developed for the study; and (4) an electrodermal activity sensor (only in MINDMAP). In HANC, the accelerometer is also used to assess the physical proximity between members of the household.

**Table 1:** Overview of the mobile sensing protocol in the MINDMAP and HANC studies

Signal monitored (corresponding device)	Measurement strategy	Validation of data
GPS (BT-Q1000XT GPS receiver, Qstarz)	One measure every 5 s, worn on the right hip for the recruitment day and 7 additional days	Daily mobility cross-validated by the travel diary and the GPS-based mobility survey
Tri-axial accelerometer (GT9X for MINDMAP and wGT3X-BT for HANC, ActiGraph)	Five-second epochs, worn on the right hip for the recruitment day and 7 additional days	Analysis of non-wear time of device
Proximity detection among household members in HANC (wGT3X-BT, ActiGraph)	Sixty-second epochs, proximity detection between nearby devices: receiver (main participant) and beacons (other household members), worn on the right hip for the recruitment day and 7 additional days	
Sleep patterns (wGT3X-BT, ActiGraph)	Sixty-second epochs, worn on the non-dominant wrist for the recruitment night and 7 additional nights (wrist-worn Actigraph not worn during the day)	Verification of bed and wake times
Electrodermal activity (EdaMove 4, movisens)	Thirty-two hertz measurement, worn on the non-dominant wrist with two adhesive electrodes attached on the hand palm during waking times for the recruitment day and 7 additional days	Analysis of nonwear time of device. Cross-validation of the physiological signal with with time-matched information on acute stress from the Eco-Emo Tracker smartphone survey
Eco-Emo Tracker smartphone survey (Samsung Galaxy S9, Android 9)	One measure every 5 s from the smartphone GPS; 8 EMA surveys per day for the recruitment day and 7 additional days	Real-time monitoring of response rates by the research assistants in the Eco-Emo Tracker web platform, permitting to intervene in time to provide support

EMA, ecological momentary assessment; GPS, Global Positioning System.

**Figure 1:**
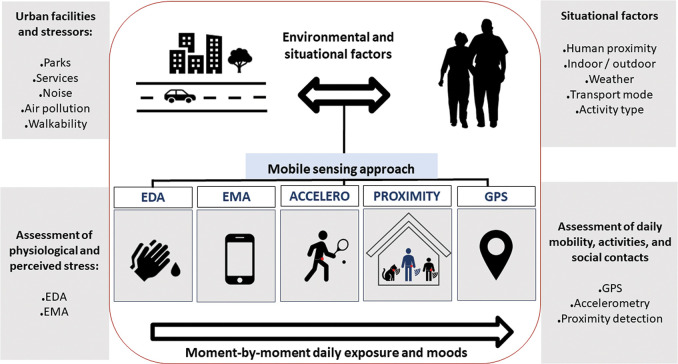
Protocol of the MINDMAP and HANC studies in RECORD. EDA, electrodermal activity; EMA, ecological momentary assessment; GPS, Global Positioning System.

Finally, based on the algorithmic processing of GPS data, a web-based mobility survey is performed to confirm, correct or complement information on places visited and transport modes used.^25^ Participants also a posteriori answered a post-questionnaire on their behaviours and mood over the 7-day period. An overview of the data collection protocol is reported in figure 1. Data collection in HANC and MINDMAP, after approval of the French Data Protection Authority, started in July 2019.

**Figure 2:**
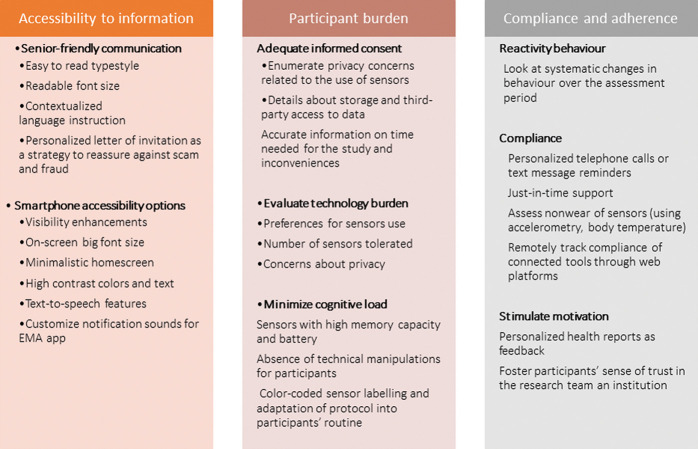
Planning, implementing and evaluating multisensors studies in older population. EMA, ecological momentary assessment.

Our double strategy to address the Hawthorne effect (behaviour change due to the protocol) is to insist with participants that they need to conduct their activities as usual and to make the protocol as convenient as possible for them to minimise reactivity behaviour related to the burden of the study, as detailed in figure 2. We do not formally measure participants’ burden but systematically take into account their feelings to ensure optimal compliance. Their main concerns are related to filling the travel diary, giving detailed information about their social network and discomfort to use the electrodermal activity (EDA) sensor because of the palm hand electrodes. However, we have consistently observed that participants mostly report concerns about the EDA device only in the first 2 days, before they get used to it (learning delay).

### Objective environmental exposures over space and time

Building on the development of GPS tracking to overcome the pitfalls of studies focused on residential neighbourhoods,^26^ our study aims to link depressive symptoms to the immediate physical and social environment of people when such feelings are experienced.

GPS-based tracking represents an opportunity to improve assessment of the exposure to built environment features but is methodologically challenging to implement. As previously reported,^10^ the selective daily mobility bias is a major source of confounding. Do places visited daily reflect actual exposure and spatial accessibility or only preferences or aversions related to unobserved intrapersonal characteristics? A recent study found the impact of selective daily mobility to be strong on the estimated association between the spatial accessibility to and use of facilities.^27^

As detailed in our previous works,^5 10 13 24^ the selective daily mobility bias is particularly strong in studies of relationships between the spatial accessibility to environmental resources and the use of such resources. This bias could also apply to mental health outcomes. For example, the association between green space exposure along the GPS trajectory (a variable of effective behaviour rather spatial accessibility) and well-being would also reflect the positive emotional effect of independent and a priori intentions that have been satisfied and realised. Our previous works have shown how to constructs mobility-based measures of spatial accessibility to resources corrected from the selective daily mobility bias.^27^

This MINDMAP and HANC protocol brings major developments for identifying the causal effect of spatial access to facilities. First, we systematically identify during our GPS-based mobility survey the destinations that are chained to another destination: indeed, it is irrelevant to calculate spatial accessibility to green spaces from the green space that was intentionally visited for the purpose of walking, but it is also irrelevant to calculate accessibility for example from the nearby café that was visited just because the green space visited (ie, that was chained to it).^24^ For the same goal of defining causally meaningful environmental exposures, participants indicate during the GPS-based mobility survey whether each destination B was on the direct way from destinations A to C, or whether a detour was made to reach B. This is useful to assess spatial accessibility to green spaces from the participant’s trip trajectory if destination B is a green space that was intentionally visited for walking.

In addition to short-term environmental exposures captured with GPS tracking and the GPS-based mobility survey, the VERITAS web mapping survey of regular destinations over the past 12 months (ie, services, friends, entertainement places, cemetery) provides information to assess exposures on a more chronic basis.^24^

Because the circumstances or situations in which people are exposed to places could modulate environmental influences, we assess with whom people travel to or spend time at places in two different ways: (1) for each of the regularly visited destinations in the VERITAS survey and (2) for each of the places visited over the GPS follow-up period during our GPS-based mobility survey. These spatialised social network data should be useful to investigate the buffering role of social support for depressive symptomatology.^12^

### Momentary assessment of mental well-being and environmental perceptions

Building on the ubiquity of smartphones in modern lives, EMA has been largely used for the momentary assessment of moods, affects and emotions in real-life settings through repeated short electronic surveys.^21^ In depression studies, EMA has been used to assess the frequency and nature of dysphoric mood states, negative affects along the day and the mental reactivity to stressors, including in samples of older persons.^28^ Using EMA, studies have explored the effect of natural outdoor environments^29^ on the momentary mood of adolescents^30^ and urban adults.^31^

In MINDMAP and HANC, each participant answers around eight EMA surveys per day at random times within predefined time slots. Four of these surveys include items of the CES-D scale; of the State-Trait Anxiety Inventory (Y-A form); and of the Vitality subscale of the 36-Item Short Form Health Survey.

In our protocol, EMA was also used to assess whether participants perceived their immediate surrounding environment as resourceful, depressing, stressful, etc, which will permit relevant comparisons between depressed and non-depressed participants. The Eco-Emo tracker application includes an algorithm based on the smartphone built-in GPS receiver that allowed us to trigger the environmental surveys only when the participant is outdoor.

Because we collect participants’ GPS data from the smartphone and from a separate GPS receiver, our study implements a geographic ecological momentary assessment (GEMA) protocol. The combination of EMA with GPS tracking^30 31^ allows us to assess all together objective built environment exposures along GPS tracks; with whom participants travel or spend time at places, which is a relevant situational predictor^12 13^ (as evaluated during the GPS-based mobility survey); and subjective perceptions of the environment, momentary mood and perceived stress from EMA. The GEMA protocol makes it possible to evaluate these objective and subjective factors through space and time dynamically along the mobility path of participants^14^ and potentially to predict how environmental features and situational factors trigger mood change in a synergistic way.^13^

### Electrodermal activity: biosensors for a continuous tracking of stress

From a biological perspective, environmental stressors can trigger a sympathetic nervous response on the skin. Sweating is an organic response to adapt the body temperature, but also a cognitive and affective reaction to stressful exposures.^32^ When the sweat glands increase their secretion activity under stress, the electrical potential and resistance of the skin changes, which can be monitored as EDA signal.^32^ For example, acute stress responses to stimuli are reflected in abrupt changes in EDA components (ie, phasic EDA, amplitude) while the emotional blunting related to depression is associated with a hypoactive EDA arousal.^15 32^

Ambulatory studies articulating EDA with depression outcomes in older adults are in their infancy.^14 16 32^ Non-laboratory monitoring of EDA permits a realistic assessment of the stress response, but many challenges remain. Internal validation and reliability are major issues in addition to technical problems including artefacts, deterioration of the electrodes and body movements affecting the physiological response.^14 32^ Due to sweating during physical exercise for the purpose of thermoregulation, physical activity is a major confounder of stress assessment from ambulatory EDA.^32^ To minimise noise during measurement, the palms of hands and feet are thought to provide more stable measures than the wrist.^32^ Individual characteristics influencing the peripheral and central nervous activity, the density of sweat glands and the use of medications are among the factors that influence EDA, and that make it challenging to analyse these data in older populations.^32^

To address these challenges, MINDMAP and HANC developed an ambulatory protocol to measure EDA over 7 days during wake hours, which is a long time of follow-up compared to existing studies. The sensor device collects the EDA signal at 32 Hz with two wet electrodes fixed on the surface of the non-dominant hand palm. The same biosensor collects time-stamped data on air pressure, temperature, acceleration and body rotation. The combination of EDA and EMA in our study provides an opportunity for cross-validating perceived stress and physiological stress in natural scenarios and for developing machine learning algorithm to predict self-reported stress from sensor signal.

## DISCUSSION

This paper presents novel methodologies to investigate how built environment features translate into stress and depressive symptoms for older person in real-life conditions. Daily mobility is a vector of exposure beyond the fixed boundaries of residential neighbourhoods in the non-residential environments where older people travel (or not).^8 10^ Understanding the interactions between personal characteristics, built environment features, and circumstances or situational factors may be useful to elucidate the mechanisms linking urban stressors to mental well-being. Such knowledge is key to develop interventions addressing these stressors or mitigating their effects.

### Alternative sensor strategies to track mood over space and time

**Figure 3:**
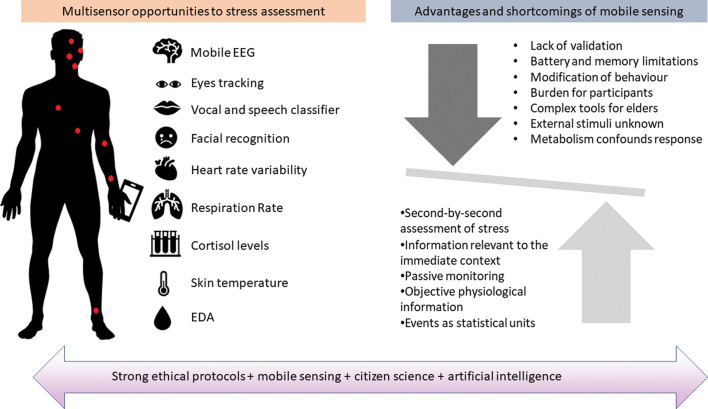
Overview of the mobile multisensor approach for studies of momentary environmental effects on mental health. EDA, electrodermal activity; EEG, electroencephalography.

The development of mobile sensing technologies offers novel opportunities to improve environmental exposure assessment and to track emotions in daily-life settings. Several biosensing techniques that are not explored in MINDMAP and HANC could provide complementary information on stress and depressive symptoms. Methods to assess emotional arousal include the measurement of heart rate and heart rate variability, respiration patterns and body temperature, as well as electromyography and electroencephalography (EEG) (figure 3).^14 16^

Mobile EEG has been recognised as an accurate and promising method in ambulatory mental health studies. The lack of ecological validity of EEG laboratory experiments led to the development of head caps, non-contact sensors on top of hair without gels and skin preparation, and ear-EEG devices for ambulatory assessment.^33^ To gain insight on mental well-being, it is low-cost and less intrusive to monitor voice and speech parameters (ie, intonation, pitch, grammar) to derive an index of stress from voice classification tools from audio record extracts.^34^ Other options to monitor stress or depressive symptoms include facial analysis and related recognition of emotions as well as mobile eye movement analysis.^35 36^ The use of log files from smartphones related to calls and short message service, screen time, application use, accelerometer and camera events may be explored to passively collect data to predict mood in everyday life.^37 38^

### Challenges of monitoring mental health in older people with passive sensors and smartphones

Even if biosensors and smartphones open an avenue for monitoring stress and depressive symptoms, there are ethical and validation challenges to the use of these data. Regarding privacy, it is critical to establish strict rules for the management and use of data and for their protection from third-party access and from the identification of study participants.^34 39^ Regarding methodological issues, the internal and external validation of biosensor data in ambulatory conditions is a priority. The development of transparent protocols is also important to ensure the replicability of studies attempting to capture the complexity of human emotions in daily-life environment. Additionally, researchers need to deal with the Hawthorne effect, to decrease participants’ modification of their habitual routines in heavy multisensor observation protocols, as alluded in figure 3.^14^ Also, importantly, these sensor studies address the short-term effect of stressors on momentary stress, anxiety and depression, but they would need to be integrated to protocols addressing the mid- to long-term psychological and biological mechanisms linking repeated exposure to stressors to the onset of mental disorders such as depression.

There are opportunities but also challenges related to the prediction of relevant psychological, behavioural or environmental information from sensor data using machine learning.^14 39 40^ Examples relevant to MINDMAP and HANC include the prediction of transport modes used from GPS and accelerometer data, or the prediction of emotions from electrodermal activity. However, the reliability of predicted variables for empirical analysis needs to be cautiously assessed.^40^ Additionally, the combination of GPS data with environmental data in a geographic information system makes it possible to assess momentary environmental exposures, but some challenges remain, for example, the difficulties to construct measures that are not vulnerable to the selective daily mobility bias^5 10^or the development of transport-mode specific exposures (eg, exposures when walking).

Overall, older people move with constraints, purposes and intentions related to their social network, preferences, needs and spatial accessibility.^8 12^ Understanding where, when, why and with whom older people move across the city will be useful to investigate the environmental and situational determinants of psychological well-being and mental health, using data that closely reflect the real experience of ageing in cities.

**What is already known on this subject**

- There is empirical evidence to suggest that chronic stress leads to depression.
- Most of the evidence about the relationship between stress resulting from environmental stimuli and depression is from laboratory experiments or short controlled experiments.
- The evidence linking built environmental characteristics to depressive symptoms in older age is mixed, and litte is known about the underlying mechanisms.
- Using static measures of contextual exposures that assign participants to their residential neighbourhood is a major limitation.

**What this study adds**

- This paper presents novel methodologies to investigate for older persons how built environment features translate into stress and depressive symptoms in real-life conditions.
- The contribution of this approach based on repeated measurements from continuous tracking includes improved personal exposure assessment, the identification of environmental and situational predictors of stress and depressive symptoms, and the detailed exploration of mechanisms.
